# Nitric oxide function during oxygen deprivation in physiological and stress processes

**DOI:** 10.1093/jxb/eraa442

**Published:** 2020-10-25

**Authors:** Isabel Manrique-Gil, Inmaculada Sánchez-Vicente, Isabel Torres-Quezada, Oscar Lorenzo

**Affiliations:** Departamento de Botánica y Fisiología Vegetal, Instituto Hispano-Luso de Investigaciones Agrarias (CIALE), Facultad de Biología, Universidad de Salamanca. C/ Río Duero 12, Salamanca, Spain

**Keywords:** Developmental cues, hypoxic stress, N-degron pathway, nitric oxide, oxygen, phytoglobins

## Abstract

Plants are aerobic organisms that have evolved to maintain specific requirements for oxygen (O_2_), leading to a correct respiratory energy supply during growth and development. There are certain plant developmental cues and biotic or abiotic stress responses where O_2_ is scarce. This O_2_ deprivation known as hypoxia may occur in hypoxic niches of plant-specific tissues and during adverse environmental cues such as pathogen attack and flooding. In general, plants respond to hypoxia through a complex reprogramming of their molecular activities with the aim of reducing the impact of stress on their physiological and cellular homeostasis. This review focuses on the fine-tuned regulation of hypoxia triggered by a network of gaseous compounds that includes O_2_, ethylene, and nitric oxide. In view of recent scientific advances, we summarize the molecular mechanisms mediated by phytoglobins and by the N-degron proteolytic pathway, focusing on embryogenesis, seed imbibition, and germination, and also specific structures, most notably root apical and shoot apical meristems. In addition, those biotic and abiotic stresses that comprise hypoxia are also highlighted.

## Teaser

Overview of nitric oxide function during plant developmental cues and stress responses when oxygen is scarce, with special emphasis on the difference between physiological hypoxia and stress-induced hypoxia.

## Introduction

Nitric oxide (NO) has important features as a key signaling molecule in plants since it is rapidly synthesized, induces defined effects within the cells, and is also scavenged quickly when no longer required.

NO is an essential component of the gaseous network described to modulate pre-adaptation to hypoxic conditions, a system that also comprises O_2_, ethylene (ET), and carbon dioxide (CO_2_) (reviewed in Sasidharan *et al.*, 2018). An optimal balance of controlled levels of reactive oxygen species (ROS) is required for plant survival. Therefore, a tightly dynamic circuit of flooding signals is essential for suitable plant responses. Diverse processes occur during this situation, such as metabolic adjustments and physiological changes, leading to plant survival. Hypoxia includes both developmental and stress-related conditions. It is important to differentiate between stress-induced hypoxia (stress hypoxia) and constitutively generated chronic hypoxia (physiological hypoxia) (Weits *et al.*, 2020). During stress hypoxia, a prompt decrease in O_2_ concentration and an NO burst occur as a result of an environmental stress (e.g. flooding), among others changes in the cellular state. This hypoxia led to different adaptive responses, mainly controlled through Group VII of the ethylene response factors (ERFVIIs). Physiological hypoxia refers to specific tissues where O_2_ concentrations are constitutively low. This type of hypoxia is found in the so-called ‘hypoxic niches’ and does not constitute a stress. Hypoxic niches have specific attributes that keep the O_2_ concentration low, including high respiration rates and the inability to release O_2_ since they are heterotrophic tissues. Among them, various growth situations are governed by lower O_2_ levels, such as embryogenesis, seed imbibition and germination, and also specific structures, most notably the root apical (RAM) and shoot apical (SAM) meristems. In addition, some biotic and abiotic stresses such as pathogen attack and flooding can lead to hypoxia.

To endure O_2_ deprivation, plants have developed sensing mechanisms, leading to transcriptional reprogramming to allow hypoxia responses. Here, we outline the influence of NO during the molecular crosstalk that underlies perception and acclimation processes. More than one source of NO is involved in the response during hypoxia, mainly nitrate reductase (NR) and plant mitochondrial activities (Gupta *et al.*, 2005; Igamberdiev *et al.*, 2005; Planchet *et al.*, 2005). The NO burst that occurs during O_2_ deprivation is not an undesirable trait and there are some data from different plant species supporting the role of NO in the plant acclimation to hypoxia (reviewed in Sasidharan *et al.*, 2018; Armstrong *et al.*, 2019).

## Nitric oxide and hypoxic stress crosstalk

**Fig. 1.**
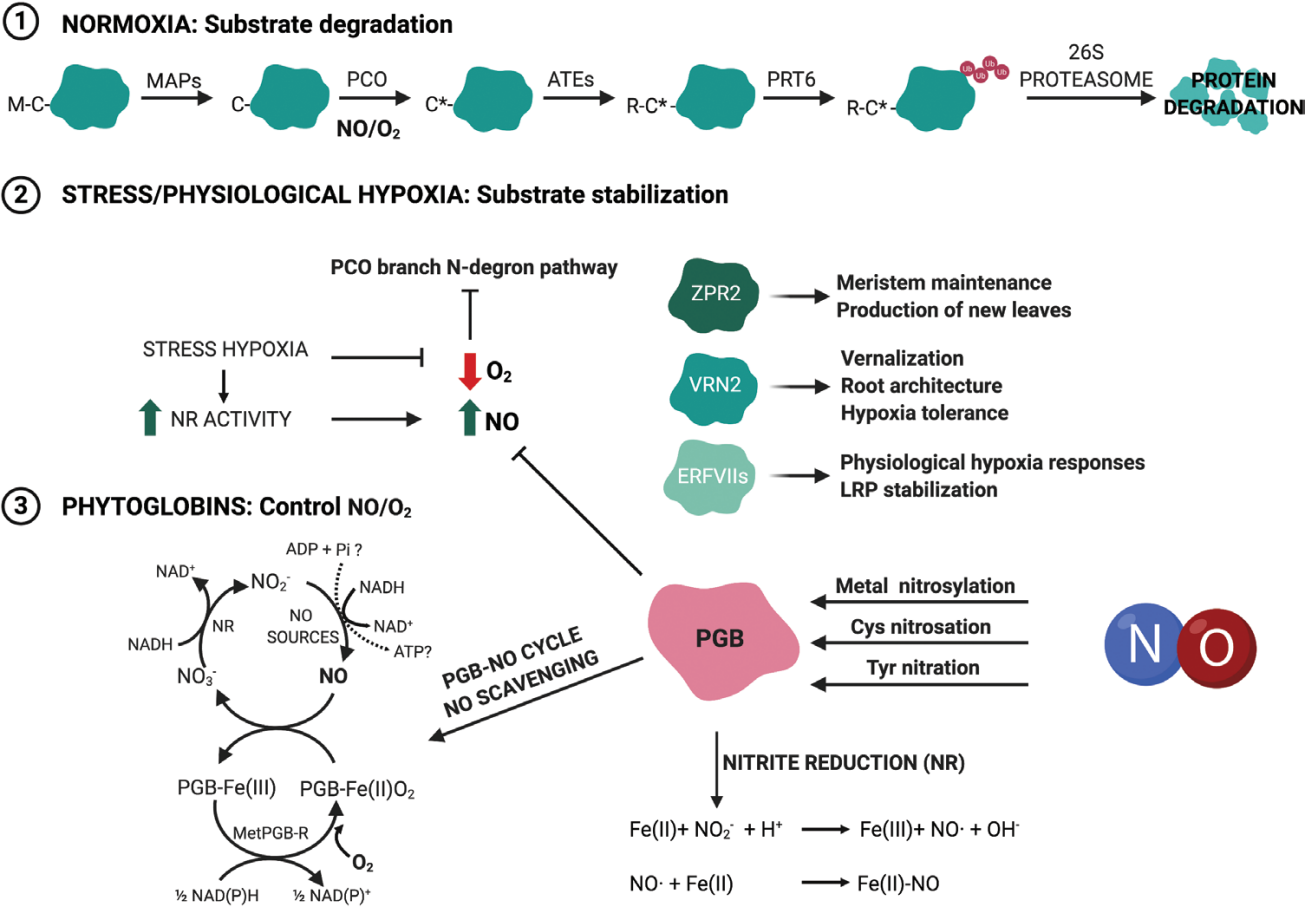
NO and O_2_ involvement in different steps of the N-degron pathway in plants and implication of PGBs. The stability of N-degron substrates is controlled by NO/O_2_ levels, whose balance is modulated by phytoglobins (PGBs). Under normoxia (1), these substrates are degraded by the action of different enzymes consecutively along the PCO branch. When plants suffer a hypoxic-related stress (2), this pathway becomes inhibited, triggering the transcriptional responses. During stress, PGBs play a key role, scavenging free NO (3), which in turn is able to modify PGBs post-translationally, to determine a finely tuned redox balance and energy status (created with BioRender.com). MAPs (methionine aminopeptidases); PCOs (plant cysteine oxidases); ATEs (arginyl-tRNA-transferases); PRT6 (PROTEOLYSIS 6); NR (nitrate reductase); ZPR2 (protein LITTLE ZIPPER 2); VRN2 (VERNALIZATION 2); ERFVII (Group VII ethylene response factors).

As aerobic organisms, plants have evolved to maintain specific requirements for O_2_ that lead to a correct respiratory energy supply. A close relationship between both O_2_ and NO sensing is mediated by phytoglobins (PGBs), which are able to modulate the level of diatomic gases such carbon monoxide (CO), NO, and O_2_, and by the N-degron pathway, which perceives the fluctuations of these gases and activates a transcriptional response through N-terminal recognition that targets proteins for degradation (Fig. 1). Hypoxic conditions lead to an increase in NO levels, suggesting a key role for the NO/O_2_ balance during this stress (Dordas *et al.*, 2003; Borisjuk *et al.*, 2007; Ma *et al.*, 2016).

### Phytoglobins modulate the balance between nitric oxide and oxygen

Maintenance of correct spatiotemporal gradients in O_2_ and NO becomes a crucial factor to determine the cellular redox status, necessary for the regulation of plant developmental and stress processes. Non-symbiotic plant hemoglobins, recently renamed phytoglobins (PGBs) (Hill *et al.*, 2016), are globular proteins able to bind small gaseous molecules such O_2_, NO, CO, and hydrogen sulfide (H_2_S). This huge binding capacity suggests an important role during sensing of gaseous molecules and regulatory mechanisms in diverse organisms from all living kingdoms, such as photosynthetic organisms, animals, fungi, or bacteria.

Hemoglobins use heme as a cofactor (Hoy and Hargrove, 2008; reviewed in Gupta *et al.*, 2011), which can bind the above-mentioned substrates, controlling their storage, transport, scavenging, and detoxification in the tissues (Arredondo-Peter *et al.*, 1998). In plants, based on sequence cladistics, three classes of PGBs exist, symbiotic (SymPGB and Lb), non-symbiotic (PGB0, 1, and 2), and truncated (PGB3) (Hoy and Hargrove, 2008). Depending on the ligands, the expression pattern and their physiological functions are categorized as symbiotic and non-symbiotic (Table 1). During stress hypoxia, caused by flooding or pathogen attack, the presence of PGBs exerts a protective role, modulating NO levels (Hartman *et al.*, 2019).

**Table 1.** Overview of the phytoglobins described in plants

Name (correlation between new and old nomenclature)	Tissue specificity	Expression pattern	Processes regulated	Binding capacity	Biophysical role	References
**Symbiotic phytoglobin (SymPhytogb)** **Symbiotic hemoglobin (sHb)**	Root nodules	Nodule-specific expression pattern	O_2_ transport and release during N_2_ fixation to maintain the flux for respiration	High affinity for O_2_	Facilitate O_2_ diffusion	Appleby *et al.* (1983); Jacobsen-Lyon *et al.* (1995); Gopalasubramaniam *et al.* (2008)
**Phytoglobin0 (Phytogb0)** **Non-symbiotic hemoglobin (nsHb)**	Whole plant	Higher expression in gametophytes; induction under hot and cold stresses, exposure to nitrate, and increased sucrose supply	NO detoxification under hypoxia stress	High affinity for O_2_	NO scavenging	Garrocho-Villegas and Arredondo-Peter (2008)
**Phytoglobin1 (Phytogb1)** **Class/type 1** **Non-symbiotic hemoglobin (nsHb-1)**	Embryonic and vegetative organs	Induction under hypoxia, ethylene, exposure to nitrate, and increased sucrose supply in roots and rosette leaves, and upon NO and H_2_O_2_ treatments	Maintenance of NO and O_2_ levels during cellular hypoxic conditions to modulate energy status	Highest affinity for O_2_, low dissociation rate	O_2_ and NO scavenging, NO dioxygenase activity	Trevaskis *et al.* (1997); Wang *et al.* (2000); Hunt et al. 2001, 2002); Lira-Ruan *et al.* (2002); Dordas et al. (2003, 2004); Perazzolli *et al.* (2004); Cantrel *et al.* (2011); Thiel *et al.* (2011); Hartman *et al.* (2019)
**Phytoglobin2 (Phytogb2)** **Class/type 2** **Non-symbiotic hemoglobin** **(nsHb-2)**	Embryonic and vegetative organs	Induction under cytokinin treatment and low temperature	Maintenance of NO and O_2_ levels during cellular hypoxic conditions and during embryogenesis; regulation of oil and sucrose accumulation in seeds	Moderate O_2_ binding capacity, low dissociation rate	O_2_ and NO scavenging, O_2_ carrier, sensing role	Trevaskis *et al.* (1997); Hunt *et al.* (2001); Dordas et al. (2003, 2004); Spyrakis *et al.* (2011); Vigeolas *et al.* (2011); Elhiti *et al.* (2018)
**Phytoglobin3 (Phytogb3)** **Class/type 3** **Non-symbiotic hemoglobin/** **truncated hemoglobin (tHb)**	Whole plant, higher in roots	Inhibition under hypoxia; induction upon auxin, NO, and H_2_O_2_ treatments and biotic stress	Modulation of NO and ROS levels during biotic stress	CO and O_2_ in a reversible manner, low O_2_ affinity	O_2_ carrier, NO dioxygenase activity	Watts *et al.* (2001); Mukhi *et al.* (2017)
**Leghemoglobin (Lb)**	Legume root nodules	Nodule-specific expression pattern	O_2_ transport and release during N_2_ fixation to maintain the flux for respiration	High affinity for O_2_	Facilitate O_2_ diffusion	Wittenberg *et al.* (1975); Hargrove *et al.* (1997); Ott *et al.* (2005)

Specifically, PGBs from Class 1 and 2 are key players at the crossroads between O_2_ and NO, since the former regulates NO turnover and the latter controls O_2_ delivery and buffering in the tissues in greater depth. These proteins are also involved in the hemoglobin–NO cycle under hypoxia, which has been proposed to relieve the inhibition of the mitochondrial transport chain by O_2_ deficiency (Dordas *et al.*, 2004; Perazzolli *et al.*, 2004; Igamberdiev *et al.*, 2005; Hebelstrup *et al.*, 2006). This cycle increases the energy status by oxidizing NAD(P)H to enhance the proton flow, resulting in ATP production. Protection against the severe effects of hypoxia depends on the binding capacity for ligands such as O_2_ or NO, since plants that overexpress a *PGB1* mutated with lower O_2_ affinity are as susceptible to hypoxia as the wild type (Hunt *et al.*, 2002). It is also proposed that PGBs 1 and 2 might function as NRs under certain conditions of extreme hypoxia (Tiso *et al.*, 2012). NO also controls PGBs post-translationally to determine a finely tuned redox balance and energy status, as will be discussed later in this review.

### The N-degron pathway operates as a nitric oxide and oxygen sensor

The plant N-degron pathway is a proteolytic system that recognizes proteins containing certain N-terminal degradation signals, called ‘N-degrons’, and polyubiquitinates them for their degradation through the 26S proteasome (Bachmair *et al.*, 1986; Varshavsky, 2011). This proteolytic pathway exists in prokaryotes and eukaryotes, and the enzyme system responsible for substrate degradation in plants is conserved with higher animals (Graciet *et al.*, 2010). In plants, there are, so far, two different N-degron pathways based on the E3 ligase, PROTEOLYSIS1 and 6 (PRT1 and PRT6), that recognize non-overlapping sets of N-terminal residues.

The PCO branch of the PRT6 N-degron pathway functions as both an O_2_ and NO sensor, as these two gases are required for the degradation of PRT6 substrates (Gibbs et al., 2011, 2014; Licausi *et al.*, 2011) (Fig. 1). Methionine-cysteine (Met-Cys-) initiating substrates undergo four enzymatic reactions prior to their degradation through the proteasome, namely Met excision (carried out by methionine aminopeptidases, MAPs), Cys oxidation (by plant cysteine oxidases, PCOs), arginylation (by arginyl-tRNA-transferases, ATEs), and polyubiquitination (by PRT6).

The O_2_ sensors in plants are thought to be the PCOs (Weits *et al.*, 2014), since these iron-dependent dioxygenases use molecular oxygen to catalyze the Cys oxidation and their *K*_m_^app^ (O_2_) values are within a physiologically relevant range for response to both external and internal O_2_ deficit that enables them to react sensitively to changes in O_2_ availability (White *et al.*, 2018). Similarly, in mammals, the ADO enzyme is required for O_2_-dependent degradation of N-degron substrates in human cells (Masson *et al.*, 2019). This enzyme, which was previously assigned as cysteamine dioxygenase, is a thiol oxidase that is functionally identical to PCOs in plants, catalyzing the conversion of the N-terminal Cys to Cys sulfinic acid. Remarkably, when human *ADO* is expressed under control of the *PCO1* promoter, it is able to complement the *pco1/2/3/4* Arabidopsis mutant and plants can develop normally. It therefore remains to be explained how NO positively influences the substrate degradation of the PCO branch of the PRT6 N-degron pathway. NO itself could affect the activities of enzymatic components of the pathway (Zarban *et al.*, 2019) or alter the cellular energy balance in an indirect manner (Armstrong *et al.*, 2019).

ERFVII group was the first substrate of the PCO branch of the PRT6 N-degron pathway described in plants (Gibbs *et al.*, 2011; Licausi *et al.*, 2011) followed by the transcriptional regulators polycomb repressive complex 2 subunit VERNALIZATION 2 (VRN2) (Gibbs *et al.*, 2018) and LITTLE ZIPPER 2 (ZPR2) (Weits *et al.*, 2019). N-degron pathway substrates regulate important aspects of plant development such as seed storage mobilization (Zhang *et al*., 2018*a*, *b*), germination (Holman *et al.*, 2009; Gibbs *et al.*, 2014), photomorphogenesis (Abbas *et al.*, 2015), stomatal closure (Gibbs *et al.*, 2014), shoot and leaf development (Graciet *et al.*, 2009), root architecture (Shukla *et al.*, 2019), SAM function (Weits *et al.*, 2019), vernalization (Gibbs *et al.*, 2018; Labandera *et al.*, 2020), flowering (Vicente *et al.*, 2017), or leaf senescence (Yoshida *et al.*, 2002), and also regulates stress responses such as flooding (Hartman *et al.*, 2019) or pathogen attack (de Marchi *et al.*, 2016; Vicente *et al.*, 2019; Till *et al.*, 2019).

In the case of VRN2 and ZPR2, these transcriptional regulators are located in hypoxic niches. ZPR2 is found in the SAM where it controls the meristem maintenance; VRN2, besides the SAM, is also located in young leaf primordia and root meristematic zones, and has a role in vernalization and root architecture (Weits *et al.*, 2019; Labandera *et al.*, 2020). The physiological hypoxia that exists in these zones prevents these proteins from degradation through the N-degron pathway.

A different regulation occurs in the case of the ERFVII group during normoxia or non-stressed growth conditions, where these transcription factors (TFs) are attached to the plasma membrane, avoiding their degradation (Licausi *et al.*, 2011). When hypoxia stress occurs (e.g. flooding), stable ERFVIIs migrate to the nucleus and activate different hypoxia response genes. When flooded, plants rapidly accumulate ET and increase the levels of the NO-scavenger PGB1. This ET-mediated NO depletion, besides hypoxia, promotes ERFVII accumulation and pre-adapts plants to survive subsequent hypoxia (Hartman *et al.*, 2019).

These results confirm the key function of the N-degron pathway in the regulation of genetic and molecular networks through NO/O_2_ balance sensing.

### Nitric oxide post-translational modifications of key hypoxia molecular players

A landmark in NO biology is the ability to modulate protein function and/or stability through three post-translational mechanisms, the nitration of Tyr residues, the *S*-nitrosation of Cys residues, and the nitrosylation of transition metals (reviewed in Sanz *et al.*, 2015; Sánchez-Vicente *et al.*, 2019b). A higher accumulation of *S*-nitrosothiols under hypoxic conditions points to this modification as a key feature by which NO exerts its responses (Hebelstrup *et al.*, 2012). *S*-Nitrosoglutathione reductase (GSNOR) is a master modulator of the intracellular levels of NO and, consequently, controls the concentration of *S*-nitrosothiols in the cell (Liu *et al.*, 2001). Autophagy constitutes an important recycling process for normal growth and also under stress conditions, including hypoxia (Chen *et al.*, 2015). It has been recently reported that NO is also coupled to hypoxia-related autophagy events through selective *S*-nitrosation of GSNOR (Zhan *et al.*, 2018). Several key molecular players during the hypoxia adaptive response are described to be controlled by NO. Previous reports indicate that this gasotransmitter inhibits cytochrome *c* oxidase (COX) (Millar and Day, 1996), aconitase (Gupta *et al.*, 2012), and ascorbate peroxidase 1 (APX1) (Begara-Morales *et al.*, 2014). Consequently, the altered enzymatic activity is reorganized to modulate O_2_ consumption, optimizing energy usage and supply.

The phytohormone ET, NO, and PGB1 are all associated with flooding-induced hypoxia since all of them are induced under O_2_ deficiency (Hebelstrup *et al.*, 2012; Hartman *et al.*, 2019). Increased NO levels are associated with NR activity under nitrite accumulation (Planchet *et al.*, 2005; Mugnai *et al.*, 2012; reviewed in Gupta and Igamberdiev, 2016). PGB1, critical for plant survival during O_2_ depletion, is also post-translationally controlled by NO through Cys nitrosation (Perazzolli *et al.*, 2004; Rubio *et al.*, 2019), metal nitrosylation (Perazzolli *et al.*, 2004), and Tyr nitration (Sainz *et al.*, 2015). Interestingly, the binding of NO to the heme group of PGBs affects the scavenging of this molecule (Gupta *et al.*, 2011). The interplay between NO and ET also impacts plant responses. Previous reports proved that both gases may affect each other, depending on the developmental stage and stress conditions studied (Magalhaes *et al.*, 2000; Li *et al.*, 2016; Liu *et al.*, 2017; Singh and Bhatla, 2018). Recently, these molecules were linked to PGB1 during flooding events, establishing a complex cycle that involved the requirement of all of them for the correct plant adaptation (Hebelstrup *et al.*, 2012; Hartman *et al.*, 2019). This overview showed us the intricate network governing hypoxia dynamic responses, mainly directed by the connection and coordination among PGB1, ET, and NO to maintain the energy state.

Abscisic acid (ABA) also participates in the response to hypoxic conditions, such as root flooding (Hsu *et al.*, 2011) or the seed environment before germination (Benech-Arnold *et al.*, 2006), and its exogenous application promotes hypoxia tolerance in roots (Ellis *et al.*, 1999). In fact, ABA perception and signaling constitute a key hormonal network affected by the N-degron pathway (Holman *et al.*, 2009; Vicente *et al.*, 2017).

## Nitric oxide impact on somatic embryogenesis and seed germination under low oxygen conditions

Somatic embryogenesis is the initiation of autonomous embryo development in somatic cells in response to exogenous and/or endogenous signals (Fehér, 2014), and is considered to be one of the most extreme examples of flexibility in plant development (Fehér *et al.*, 2003). The phases of somatic embryogenesis as a morphogenic phenomenon are characterized by distinct biochemical and molecular events (Suprasanna and Bapat, 2005). The first phase is the induction stage in which differentiated somatic cells acquire embryogenic competence. This phase is followed by the expression or initiation of somatic embryogenesis in which competent cells or proembryos start developing. Finally, during maturation, somatic embryos anticipate germination by desiccation and accumulation of reserves (Jiménez, 2001).

**Fig. 2.**
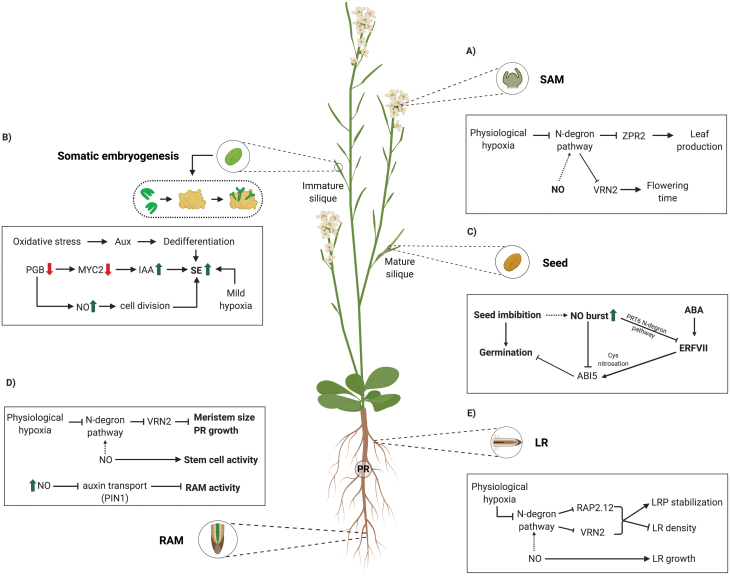
Network of NO and low oxygen interactions in a developmental stage-based context. Somatic embryogenesis, seed germination, RAM, and SAM. (A) The SAM displays a state of physiological hypoxia which prevents N-degron pathway activation, that is also influenced by low NO levels. VRN2 contributes to vernalization and hypoxia tolerance, while ZPR2 sustains leaf production in the SAM. (B) SE is generally favored by mild hypoxia, and oxidative stress-inducing compounds promote dedifferentiation by increasing endogenous auxin levels. NO stimulates the activation of cell division and embryogenic cell formation in some systems. Mutation of *PGB2* increases the number of somatic embryos by suppressing the expression of *MYC2* and induces the transcription of several IAA biosynthetic genes promoting SE. (C) NO is necessary for completion of germination; NO binds to ABI5, through Cys *S*-nitrosation of Cys153, and promotes the interaction with CULLIN4-based and KEEP ON GOING E3 ligases and consequently its degradation by the proteasome. *ABI5* is modulated by NO and O_2_ through the N-degron pathway. Members of the ERFVII group have been identified as *ABI5* transcriptional activators. (D) NO is necessary for normal RAM organization; however, high levels of NO reduce PIN1-dependent auxin transport, reducing RAM activity. NO influences meristem size and promotes PR root growth by preventing N-degron pathway activation. (E) NO donor treatments promote lateral root growth in a dose-dependent manner, NO could be promoting RAP2.12 degradation and thus reducing LRP stabilization and inhibiting LR density. Arrows and bars indicate positive and inhibitory effects, respectively. Dotted arrows and bars indicate putative regulations (created with BioRender.com). ZPR2 (protein LITTLE ZIPPER 2); VRN2 (VERNALIZATION 2); PGB2 (phytoglobin 2); MYC2 (basic helix–loop–helix protein 6); IAA (indole-3-acetic acid); ABA (abscisic acid); ABI5 (ABA INSENSITIVE 5); ERFVII (Group VII ethylene response factors); PRT6 (PROTEOLYSIS 6); PIN1 (PIN-FORMED 1); SAM (shoot apical meristem); SE (somatic embryogenesis); RAM (root apical meristem); PR (primary root); LR (lateral root).

Two categories of inductive conditions which allow differentiated cells to develop into competent dedifferentiated cells are now recognized. These include plant growth regulators and stress factors (reviewed in Zavattieri *et al.*, 2010). It has been described that this process is generally favored by mild hypoxia (Thorpe and Stasolla, 2001), which mimics the low O_2_ environment accompanying zygotic embryo development (Rolletschek *et al.*, 2003) (Fig. 2).

An increasing number of publications link ROS and somatic embryogenesis. Oxidative stress-inducing compounds increase the cell endogenous auxin levels and promote dedifferentiation (Pasternak *et al.*, 2002; Correa-Aragunde *et al.*, 2006). Ötvös *et al*. (2005), working with alfalfa cell cultures, showed that H_2_O_2_ and NO have a promoting effect on somatic embryogenesis. NO stimulates the activation of cell division and embryogenic cell formation in leaf protoplast cells of alfalfa in the presence of auxins.

In Arabidopsis, PGB1 scavenges NO produced under severe hypoxia, thus fulfilling a protective role during stress conditions (Dordas *et al.*, 2003, 2004; Perazzolli *et al.*, 2004). Like *PGB1*, overexpression of *PGB2* enhances survival under hypoxic conditions through removal of cellular NO (Hebelstrup *et al.*, 2006, 2012; Hebelstrup and Jensen, 2008). Mutation of *PGB2* increases the number of Arabidopsis somatic embryos by suppressing the expression of *MYC2*, a repressor of auxin synthesis, and inducing the transcription of several indole-3-acetic acid (IAA) biosynthetic genes (Elhiti *et al.*, 2013). An experimental reduction of NO through pharmacological treatments reverses the effects of *PGB2* suppression on somatic embryogenesis (Elhiti *et al.*, 2013). This phenotype can be reversed by the re-introduction of PGB2 in the nucleus but not in the cytoplasm; this promotive effect can be attenuated by reducing the level of NO (Godee *et al.*, 2017).

Embryo production in Arabidopsis appears to be susceptible to NO levels, as it is increased in the presence of the NO donors sodium nitroprusside (SNP) and *S*-nitroso-*N*-acetylpenicillamine (SNAP) and is decreased after scavenging with 2-phenyl-4,4,5,5,-tetramethylimidazoline-1-oxyl 3-oxide (PTIO) and carboxy-PTIO (Elhiti *et al.*, 2013). Hypoxia is also linked to non-stress conditions, but at specific developmental stages such as seed imbibition and germination. The outermost layers of seed restrict O_2_ diffusion, leading to hypoxic or even almost anoxic states of inner seed tissues (Borisjuk *et al.*, 2007). NO accumulation in response to O_2_ deficiency was described, avoiding endogenous anoxia and fermentation (Borisjuk *et al.*, 2007). This gasotransmitter mediates a reversible O_2_ balance through modulation of respiratory fluxes, facilitating energy supply for the synthesis of storage compounds. *PGB1* and *2* overexpression also promotes the metabolic reprogramming and lower NO content in the seed (Thiel *et al.*, 2011; Vigeolas *et al.*, 2011), highlighting again the importance of the molecular team composed of O_2_, NO, and PGBs.

NO burst was also described during early seed germination events (Simontacchi *et al.*, 2004; Albertos *et al.*, 2015). This NO free gas is absolutely necessary for completion of germination at different molecular levels, converging into the bZIP TF ABI5 (reviewed in Sánchez-Vicente *et al.*, 2019a, b). This TF represents a molecular hub during germination repression mediated by ABA (Finkelstein and Lynch, 2000; Lopez-Molina *et al.*, 2001). NO binds directly to ABI5, through Cys *S*-nitrosation of Cys153, promoting the interaction with CULLIN4-based and KEEP ON GOING E3 ligases and consequently its degradation by the proteasome (Albertos *et al.*, 2015). Additional post-translational levels of ABI5 regulation by NO correspond to the SUMO E3 ligase SIZ1, which is considered a Tyr nitration target (Lozano-Juste *et al.*, 2011), and to the SNF1-RELATED PROTEIN KINASE2 (SnRK2), whose activity is inhibited by *S*-nitrosation (Wang *et al.*, 2015). At the transcriptional level, *ABI5* is also modulated by NO and O_2_ through the N-degron pathway. Members of the ERFVII group were identified as *ABI5* transcriptional activators (Gibbs *et al.*, 2014). Additionally, the ERFVII group controls the *ABI5* transcriptional repressor BRAHMA (Vicente *et al.*, 2017).

The network integrated by NO, O_2_, and PGBs tightly regulates ABI5, at both the transcriptional and post-translational levels, highlighting the fine-tuning mechanisms controlling early developmental stages, which are governed by low O_2_ abundance.

## Nitric oxide function in the RAM and SAM, locations with scarce oxygen concentration

Meristems are populations of small, isodiametric cells with embryonic characteristics. Vegetative meristems are self-perpetuating; not only do they produce all tissues and organs, but they also retain their embryonic character indefinitely (Taiz *et al.*, 2014). Previous studies have measured and defined the O_2_ concentration profile in the maize RAM (Gibbs *et al.*, 1998; Darwent *et al.*, 2003) and, more recently, Weits *et al.* (2019) shaped the O_2_ profile in the Arabidopsis SAM, using a micro-scale Clark-type oxygen sensor. Both meristems display a decrease in O_2_ concentration in the central zone, the area committed to the maintenance of the stem cells that sustain growth and development.

Besides O_2_ levels, NO has an important role in the maintenance of the meristems, and alteration in NO homeostasis is sufficient to influence the fate of whole meristems. NO is necessary for normal RAM organization (Sanz *et al.*, 2014); however, high levels of NO reduce auxin transport via a PIN1-dependent mechanism and RAM activity is reduced concomitantly (Fernández-Marcos *et al.*, 2011; Sanz *et al.*, 2014).

Some substrates of the N-degron pathway are found in meristems, where they have important functions (Gibbs *et al.*, 2018; Weits *et al.*, 2019; Labandera *et al.*, 2020). The physiological hypoxia that exists in the meristems prevents its degradation through the N-degron pathway. During hypoxia, NO levels must be also kept low to prevent N-degron pathway activation. ZPR2 sustains leaf production in the SAM (Weits *et al.*, 2019), and VRN2 is found in the SAM, RAM, and lateral root primordia (LRPs) where it contributes to vernalization (cold-induced flowering) and hypoxia tolerance (Gendall *et al.*, 2001; Gibbs *et al.*, 2018). In LRPs, stabilized RAP2.12 (a member of ERFVII group) induces expression of core hypoxia-responsive genes, promoting LRP stabilization by attenuating auxin signaling (Shukla *et al.*, 2019). Remarkably, there is a differential gene regulation between LRPs and the RAM since these hypoxia-responsive genes are not expressed in the RAM. According to this, NO donor treatments promote lateral root growth in a dose-dependent manner, while primary root growth is arrested (Correa-Aragunde *et al.*, 2004). In LRPs, NO could be promoting RAP2.12 degradation and thus reducing LRP stabilization.

**Fig. 3.**
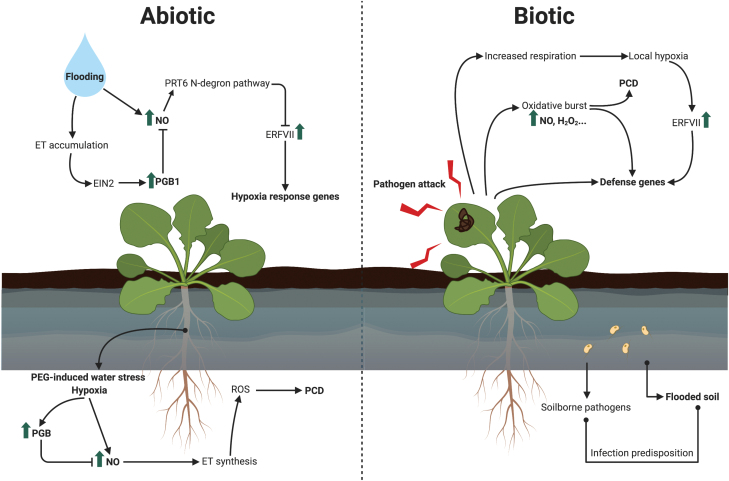
Network of NO and low oxygen interactions in a stress-based context. Abiotic and biotic stresses. Left: abiotic stress such as flooding causes NO and ethylene (ET) accumulation. ET signaling promotes enhanced levels of the NO-scavenger phytoglobin 1 (PGB1), limiting Group VII ethylene response factor (ERFVII) degradation through inactivation of the PRT6 N-degron pathway. ERFVII members induce expression of core hypoxia response genes. In roots, PGBs also protect meristems during PEG-induced water stress and hypoxia, since they scavenge NO to prevent programmed cell death (PCD), a process initiated by the overaccumulation of NO and mediated by ET and ROS. Right: after certain pathogen attacks, the respiration rate increases in the plant, leading to local hypoxia that promotes accumulation of ERFVII members. The oxidative burst (ROS and NO) as a response to the infection can trigger PCD and promotes, along with ERVII members, activation of defense genes. In addition, flooded soils predispose root plants to infections by soilborne pathogens (created with BioRender.com). EIN2 (ETHYLENE INSENSITIVE 2); PGB1 (phytoglobin 1); ERFVII (Group VII ethylene response factors); ROS (reactive oxygen species); PCD (programmed cell death).

*PGB* gene expression patterns in meristems (Heckmann *et al.*, 2006; Hebelstrup *et al.*, 2007, 2012) may indicate that these proteins are facilitating, alongside hypoxia, the stabilization of N-degron pathway substrates by reducing NO levels. PGBs also have a central role in the protection of meristems during stress, specifically the RAM. This meristem is particularly susceptible to environmental perturbations (e.g. salinity, drought, and flooding) since it is directly exposed to the soil. NO overaccumulates at the root tip under stress (Fernández-Marcos *et al.*, 2011; Liu *et al.*, 2015), risking RAM functionality. High levels of NO increase ET production to inhibit meristematic cell proliferation and to induce cell death through ROS (Mira *et al.*, 2016*b*). PGBs have been reported to protect meristems during polyethylene glycol (PEG)-induced water stress (Mira *et al.*, 2017) and hypoxia (Mira *et al.*, 2016*b*) (Fig. 3). Under these conditions, PGBs accumulate to reduce the programmed cell death (PCD) initiated by the high levels of NO and mediated by ET via ROS (Mira *et al.*, 2016*b*, 2017). In addition, plants with jeopardized *PGB1* gene expression show a number of shoot- and leaf-related phenotypes that include flowering delay, the tendency of the SAM to reverse from the bolting stage to the rosette stage (Hebelstrup and Jensen, 2008), and stunted leaves with enlarged hydathodes (Hebelstrup *et al.*, 2006). These phenotypes are coincident with NO accumulation in the affected organs, which hints at a role for PGBs in modulation of NO signaling during plant development (Hebelstrup *et al.*, 2007).

## Role of nitric oxide signaling between low oxygen and biotic stress

Plants rely on a sophisticated network of signal transduction pathways to respond to pathogen attacks and unfavorable environmental conditions, which leads to metabolic and transcriptional reprogramming (Valeri *et al.*, 2020). Several phytohormones have been related to plant defense, among them salicylic acid (SA) is predominantly associated with biotrophs, while jasmonic acid (JA) and ET are associated with necrotrophs (Wildermuth *et al.*, 2001; Thaler *et al.* 2004; reviewed in Conrath, 2006; Halim *et al*.. 2006).

Although NO in plants is being revealed to be involved in a great variety of cellular processes associated with growth and development (reviewed in Sanz *et al.*, 2015), it was first described as a molecule involved in the plant immune response (Delledonne *et al.*, 1998; Durner *et al.*, 1998). Basal defenses and hypersensitive responses rely on NO (Mur *et al.*, 2013). For instance, modulation of *Pgb* expression, which is naturally up-regulated by low oxygen tensions (Taylor *et al.*, 1994; Hunt *et al.*, 2002), has been shown to influence plant responses to a variety of pathogens, and suppression of *Pgb* resulted in elevated levels of NO, hydrogen peroxide, and JA in Arabidopsis plants infected with *Botrytis cinerea* (Mur *et al.*, 2012; reviewed in Mira *et al.*, 2016a).

One of the earliest cellular responses following successful pathogen recognition is the so-called oxidative burst, which is a rapid, transient production of ROS via consumption of O_2_, that can trigger hypersensitive cell death (Wojtaszek, 1997; Govrin and Levine, 2000; Torres *et al.*, 2006). In this context, it is difficult to separate NO from ROS, considering that their signaling pathways in plant biotic interactions are closely connected (Scheler *et al.*, 2013; reviewed in Sánchez-Vicente *et al.*, 2019b).

NO also plays a major role in the signaling pathways of phytopathogenic fungi. For instance, the expression of the *B. cinerea* flavohemoglobin gene (*Bcfhg1*), which is the main NO detoxification method in this fungus, is developmentally regulated, with peak expression levels during germination of conidia, and is enhanced very quickly upon exposure to NO of germinating conidia. It is believed that the production of NO by *B. cinerea* is probably modulated to promote fungal colonization of the plant tissue (Turrión-Gómez *et al*., 2010; Turrión Gómez and Benito, 2011). Furthermore, the application of external NO to *Colletotrichum coccodes* defers spore germination, whilst treatment with NO scavengers stimulates spore germination (Wang and Higgins, 2005).

Moreover, low O_2_ predisposes plants to infection by soilborne pathogens (Fig. 3). For instance, oxygen-deficient soils stress plants and predispose them to infection by water molds such as *Pythium* and *Phytophthora cinnamomi* (Davison *et al.* 1993), and O_2_-deprived roots leak greater amounts of soluble metabolites and ethanol, attracting zoospores (Kozlowski, 1997; Badri and Vivanco, 2009). Thus, as an aerobic organism and in a water-saturated growing medium, *P. cinnamomi* zoospores will infect roots near the surface where there is enough O_2_.

The inactivation of different components of the Arg/N-degron pathway results in greater susceptibility of Arabidopsis to necrotrophic pathogens. Thus, it has been shown that induction of components of the hypoxia response, controlled by the ERFVIIs, enhanced clubroot disease progress, indicating that the protist hijacks the N-end rule ERFVII regulation system to enhance infection (Gravot *et al.*, 2016). Early studies indicate that RAP2.3, and maybe other ERFVII TFs, might be key regulators in both the low-oxygen and plant biotic stress responses (Valeri *et al.*, 2020). The results of Kim *et al.* (2018) show that OCTADECANOID-RESPONSIVE ARABIDOPSIS 59 (ORA59), one of the best characterized ERF TFs involved in *B. cinerea* resistance, interacts with RAP2.3, and its expression is induced synergistically by JA and ET, confirming its importance in the JA and ET signaling pathway (Pré *et al.*, 2008). In this regard, Arabidopsis plants overexpressing *RAP2.2* and a mutant line showed higher resistance and more susceptibility, respectively, suggesting an important role for RAP2.2 against the infection by the necrotroph (Zhao *et al.*, 2012). A recent study conducted by Valeri *et al.* (2020) indicates that infection by *B. cinerea* induces increased respiration, leading to a drastic drop in the O_2_ level in the leaf and that the establishment of this local hypoxic area results in stabilization and nuclear relocalization of RAP2.12. As a consequence, this nuclear relocalization activates the hypoxia-responsive gene network, implying that ERFVII proteins can become stabilized in infected tissue and have an influence in pathogen resistance, allowing RAP2.3 to form a complex with ORA59 to regulate plant defense genes (Kim *et al.*, 2018) or influencing other proteins with a hypoxia-dependent stabilization.

## Concluding remarks

Among the challenges imposed by global warming, the forecast of unexpected and increased floods will cause limitations in plant normal development and productivity for agricultural purposes. Therefore, the control of plant responses to this hypoxia scenario is a landmark aspect for future research, as it critically impacts on seed germination, plant development and establishment, and, consequently, on plant productivity. The identification of the elements and the molecular bases that participate in hypoxic stress responses is essential to understand their function in the plant, which is a prerequisite for its genetic improvement. Thus, advances in the study of plant priming using NO-related compounds to enhance hypoxia tolerance could be achieved in a similar way to ET and ABA pre-treatments (Ellis *et al.*, 1999; Hartman *et al.*, 2019).

In parallel, this environmental modification can favor the development of new plant pests and pathogens or increase the incidence levels of those that exist today. Nowadays some controversy still surrounds the NO homeostasis in plant immunity, at the level of both production and turnover (reviewed in Vandelle *et al.*, 2016), that needs to be solved for a better pest control.

The N-degron pathway was identified as a new NO sensor that functions through its ability to destroy specific regulatory proteins bearing N-terminal Cys residues in mammals (Hu *et al.*, 2005; Masson *et al.*, 2019). In plants, apart from the evidence reported by Gibbs *et al.* (2014, 2018) on the proteolytic control of ERFVII group of TFs and polycomb repressive complex 2 subunit VRN2, respectively, no other target has been related to NO directly. Deciphering the mechanism of NO sensing, by direct binding of the molecule, and the post-translational regulation of molecular targets across the different components of the N-degron pathway will shed light on controlling hypoxia, which is detrimental for plant survival.
